# Title, Table of Contents and Acknowledgements

**DOI:** 10.1080/26410397.2021.2022896

**Published:** 2022-01-25


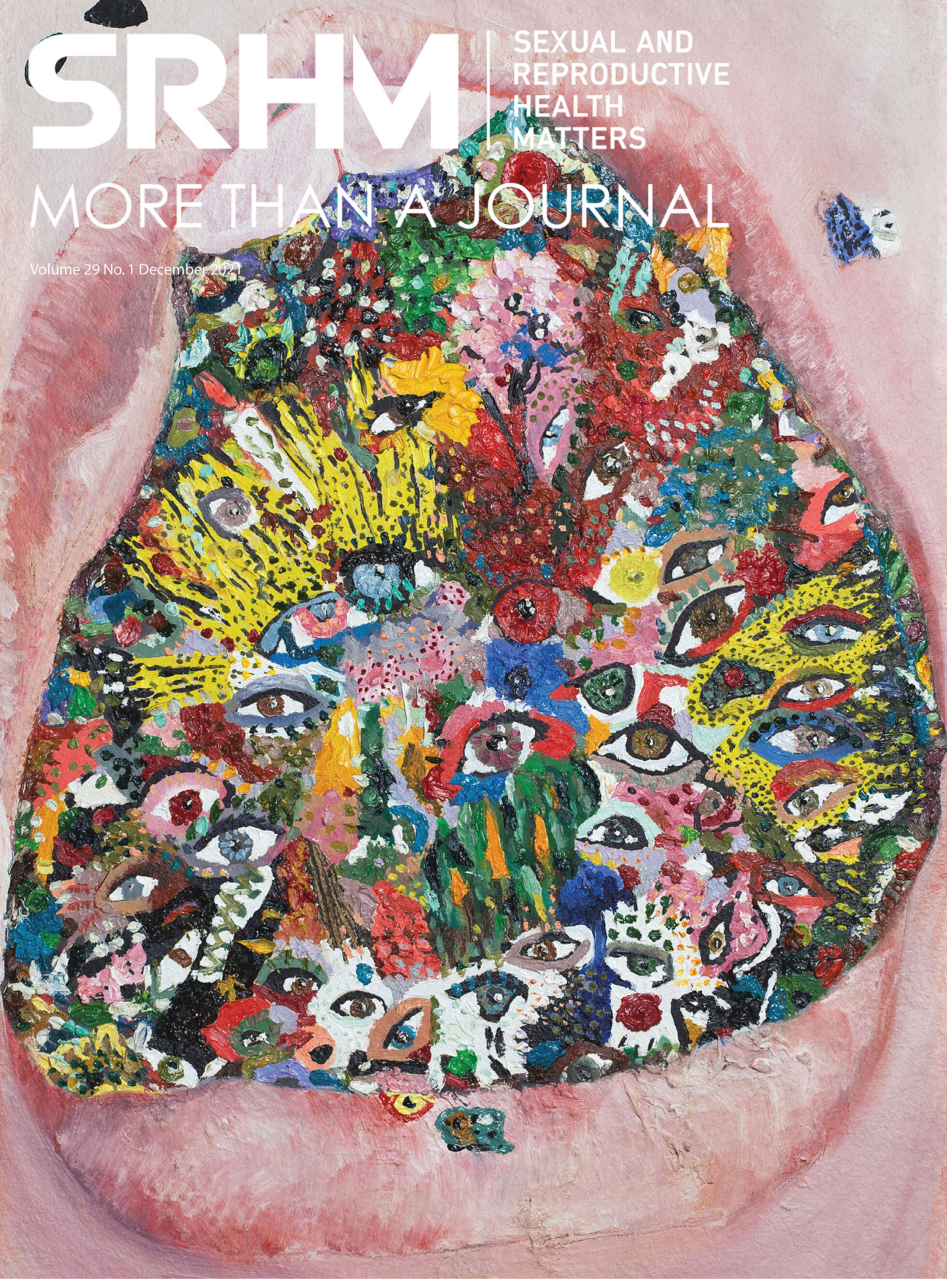


**Editorial**

1 *Emma Pitchforth, Julia Hussein* Moving ahead together, on a foundation of rights-based evidence

**Commentaries**

5 *Anthony Idowu Ajayi, Ramatou Ouedraogo, Kenneth Juma, Grace Kibunja, Collins Cheruiyot, Meggie Mwoka, Emmy Kageha Igonya, Winnie Opondo, Emmanuel Otukpa, Caroline W. Kabiru, Boniface Ayanbekongshie Ushie* Research priorities to support evidence-informed policies and advocacy for access to safe abortion care in sub-Saharan Africa

9 *Alexandria K. Mickler, Maria A. Carrasco, Laura Raney, Vinit Sharma, Ados V. May, Jennie Greaney* Applications of the High Impact Practices in Family Planning during COVID-19

18 *Catalina Martínez Coral, Carmen Cecilia Martínez* Sexual violence against girls in schools as a public health issue: a commentary on the case *Paola Guzmán Albarracín v. Ecuador*

23 *Lucía Berro Pizzarossa, Rishita Nandagiri* Self-managed abortion: a constellation of actors, a cacophony of laws?

31 *Julie Hennegan, Inga T. Winkler, Chris Bobel, Danielle Keiser, Janie Hampton, Gerda Larsson, Venkatraman Chandra-Mouli, Marina Plesons, Thérèse Mahon* Menstrual health: a definition for policy, practice, and research

39 *Sharon Bissell, Erin Sines, Dipa Nag Chowdhury, Kole Shettima* From population control to reproductive health and rights: a donor’s journey: *The John D and Catherine T MacArthur Foundation’s population and reproductive health program, 1986–2019*

**Review articles**

44 *Lucy C Wilson, Kate H Rademacher, Julia Rosenbaum, Rebecca L Callahan, Geeta Nanda, Sarah Fry, Amelia C L Mackenzie* Seeking synergies: understanding the evidence that links menstrual health and sexual and reproductive health and rights

57 *Madina Agénor, Gabriel R. Murchison, Jesse Najarro, Alyssa Grimshaw, Alischer A. Cottrill, Elizabeth Janiak, Allegra R. Gordon, Brittany M. Charlton* Mapping the scientific literature on reproductive health among transgender and gender diverse people: a scoping review

75 *Roopan K. Gill, Amanda Cleeve, Antonella F. Lavelanet* Abortion hotlines around the world: a mixed-methods systematic and descriptive review

90 *Andrea Hannah Kaiser, Björn Ekman, Madeleine Dimarco, Jesper Sundewall* The cost-effectiveness of sexual and reproductive health and rights interventions in low- and middle-income countries: a scoping review

104 *Anna Kågesten, Miranda van Reeuwijk* Healthy sexuality development in adolescence: proposing a competency-based framework to inform programmes and research

**Research articles *Abortion***

121 *Samantha Chareka, Tamaryn L. Crankshaw, Pemberai Zambezi* Economic and social dimensions influencing safety of induced abortions amongst young women who sell sex in Zimbabwe

133 *Tiziana Leone, Ernestina Coast, Sonia Correa, Clare Wenham* Web-based searching for abortion information during health emergencies: a case study of Brazil during the 2015/2016 Zika outbreak

146 *Ann M. Moore, Juliette Ortiz, Nakeisha Blades, Hannah Whitehead, Cristina Villarreal* Women’s experiences using drugs to induce abortion acquired in the informal sector in Colombia: qualitative interviews with users in Bogotá and the Coffee Axis

162 *Lianne Holten, Eva de Goeij, Gunilla Kleiverda* Permeability of abortion care in the Netherlands: a qualitative analysis of women’s experiences, health professional perspectives, and the internet resource of Women on Web

180 *Leen De Kort, Edwin Wouters, Sarah Van de Velde* Obstacles and opportunities: a qualitative study of the experiences of abortion centre staff with abortion care during the first COVID-19 lockdown in Flanders, Belgium

196 *Jill Durocher, Catherine Kilfedder, Laura J. Frye, Beverly Winikoff, Karthik Srinivasan* A descriptive analysis of medical abortion commodity availability and pricing at retail outlets in 44 countries across four regions globally

214 *Céline Miani* Medical abortion ratios and gender equality in Europe: an ecological correlation study

**Adolescents and young people**

232 *Yvette Ruzibiza, Lidewyde Berckmoes, Stella Neema, Ria Reis* Lost in freedom: ambivalence on sexual freedom among Burundian adolescents living in the Nakivale refugee settlement, Uganda

246 *Alexandra M. Minnis, Emily Krogstad, Mary Kate Shapley-Quinn, Kawango Agot, Khatija Ahmed, L. Danielle Wagner, Ariane van der Straten, on behalf of the TRIO Study Team* Giving voice to the end-user: input on multipurpose prevention technologies from the perspectives of young women in Kenya and South Africa

261 *Katherine Watson, Elsie Akwara, Patricia Machawira, Maria Bakaroudis, Renata Tallarico, Venkatraman Chandra-Mouli* The East and Southern Africa Ministerial Commitment: a review of progress toward fulfilling young people's sexual and reproductive health and rights (2013–2018)

287 *Ruba Al Akash, Morgen A. Chalmiers* Early marriage among Syrian refugees in Jordan: exploring contested meanings through ethnography

**Family planning and contraception**

303 *Batula Abdi, Jerry Okal, Gamal Serour, Marleen Temmerman* Muslim men’s perceptions and attitudes on family planning: a qualitative study in Wajir and Lamu counties in Kenya

314 *Euphemia Sibanda, Ania Shapiro, Bradley Mathers, Annette Verster, Rachel Baggaley, Mary E. Gaffield, Virginia Macdonald* Values and preferences of contraceptive methods: a mixed-methods study among sex workers from diverse settings

336 *Katherine Tumlinson, Laura E. Britton, Caitlin R. Williams, Debborah Muthoki Wambua, Dickens Otieno Onyango* Informal payments for family planning: prevalence and perspectives of women, providers, and health sector key informants in western Kenya

353 *Theresa Nkole, Adam Silumbwe, Margarate N. Munakampe, Joanna Paula Cordero, Cecilia Milford, Joseph Mumba Zulu, Petrus S. Steyn* Community and health provider perspectives on the quality of family planning and contraceptive services in Kabwe District, Zambia

**Other topics in sexual and reproductive health and rights**

367 *Sofia Gruskin, William Jardell, Laura Ferguson, Kristin Zacharias, Rajat Khosla* Integrating human rights into sexual and reproductive health research: moving beyond the rhetoric, what will it take to get us there?

377 *Marcin Smietana, Sharmila Rudrappa, Christina Weis* Moral frameworks of commercial surrogacy within the US, India and Russia

394 *May Sudhinaraset, Katie Giessler, Michelle Kao Nakphong, Kali Prosad Roy, Ananta Basudev Sahu, Kovid Sharma, Dominic Montagu, Cathy Green* Can changes to improve person-centred maternity care be spread across public health facilities in Uttar Pradesh, India?

409 *Julie Hennegan, Funmilola M. OlaOlorun, Sani Oumarou, Souleymane Alzouma, Georges Guiella, Elizabeth Omoluabi, Kellogg J. Schwab* School and work absenteeism due to menstruation in three West African countries: findings from PMA2020 surveys

425 *Lashanda Skerritt, Alexandra de Pokomandy, Nadia O’Brien, Nadia Sourial, Ann N Burchell, Gillian Bartlett, Tibor Schuster, Danielle Rouleau, Karène Proulx-Boucher, Neora Pick, Deborah Money, Rebecca Gormley, Allison Carter, Mark H Yudin, Mona Loutfy, Angela Kaida, CHIWOS Research Team* Discussing reproductive goals with healthcare providers among women living with HIV in Canada: the role of provider gender and patient comfort

441 *Jasmine Sprague Hepburn, Idil Shekh Mohamed, Björn Ekman, Jesper Sundewall* Review of the inclusion of SRHR interventions in essential packages of health services in low- and lower-middle income countries

453 *Lucila Szwarc, Victoria Sánchez Antelo, Melisa Paolino, Silvina Arrossi* “I’m neither here, which would be bad, nor there, which would be good”: the information needs of HPV+ women. A qualitative study based on in-depth interviews and counselling sessions in Jujuy, Argentina

**Perspectives**

464 *Tomoko Suga* Protecting women: new domestic violence countermeasures for COVID-19 in Japan

467 *Katherine J. Kramer, Aliye Runyan, Elizabeth A. Micks, Sejal Tamakuwala, Mary Reid, Suha Syed, Conrad R. Chao, Maurice-Andre Recanati* Unequal medicine harms: reflections on the experiences of an intersex physician

**Editor-in-Chief:** Julia Hussein (to March)

**Executive Editor**: Emma Pitchforth (from April)

**Chief Executive**: Eszter Kismödi

**Senior Editors**: Sarah Keogh, TJ Sundari Ravindran

**Managing Editor**: Pete Chapman

**Monitoring Editor**: Pathika Martin

**South Asia Hub Manager**: Sanjeeta Gawri

**Communications Manager**: Alexane Bremshey

**Operations Manager**: Amy Griffiths

**Associate Editors:** Laura Ferguson, Atsumi Hirose, Nambusi Kyegombe, Helen Potts, Mindy Jane Roseman, Nina Sun, Joyce Wamoyi

**Funding:** SRHM's work in 2021 has been supported by the Bill and Melinda Gates Foundation and the Open Society Foundations.

**Cover image:** “A Certain Blindness” by Grace Cross.

**Translation:** Françoise de Luca-Lacoste translated abstracts from English to French and Lisette Silva translated abstracts from English to Spanish.

**Copyright © 2022 Sexual and Reproductive Health Matters.** This is an Open Access journal distributed under the terms of the Creative Commons Attribution License (http:// creativecommons.org/licenses/ by/4.0/), which allows for sharing and adapting the work for any purpose, even commercially, provided appropriate credit is given with a link to the originally published item, a reference to the author(s) and links to their homepages, reference to the license under which the article is published and a link to this, as well as an indication of any changes that have been made to the original. ISSN (Online) 2641-0397

**Peer reviewers:**

Claudia Abreu Lopes, Timothy Osebe Abuya, Pranita Achyut, Tahera Ahmed, Carolyne Ajema, James Akazili, Elsie Akwara, Cristina Alonso, Ilana Ambrogi, Joe Amon, Lise Ulrik Andreasen, Samuel Kojo Antobam, Subha Sri B, Finley Baba, Rob Bain, Barbara Baird, Aduragbemi Banke-Thomas, Suchi Bansai, Heidi Bart Johnson, Alka Barua, Suzanne Bell, Gervais Beninguissé, Tshegofatso Phalane Bessenaar, Nandiat Bhan, Lekha D Bhat, Ann Biddlecom, Antonia Biggs, Kelly Blanchard, Nina Brooks, Shyam Sundar Budhathoki, Abigail Burgess, Lisa Caruana-Finkel, Lidia Cecilia Casas, Ishita Chatterjee, Sreeparna Chattopadhyay, Sylvester C Chima, Megan Christofield, Carly A Comins, Bergen Cooper, Jane Cover, Suchitra Dalvie, Shrinivas Satyanarayan Darak, Jashodhara Dasgupta, Jocelyn DeJong, Preeti Dhillon, Farah Diaz-Tello, Catherine Dodds, Soo Downe, Ilana Dzuba, Myles Elledge, Joanna Erdman, Fatima Estrada, Sandra Fernández, Katherine Footman, Barbara Friedland, Katherine Gambir, Samantha Garbers, Rakhi Ghoshal, Roopan Gill, Srinivas Goli, Meena Gopal, Arne Gouwy, Yufu Iguchi, Katja Isaksen, Olena Ivanova, Sharad D Iyengar, Heather Jacobson, Vathsala Jaysuriya-Illesinghe, Dyah Juliastuti, Shveta Kalyanwala, Shanna Katz Kattari, Talat Khadivzadeh, Renu Khanna, Andrea Gael Kinnear Wilson, Abhay Kudale, Ekaterina Kulchavenya, Bhavita Kumari, Sara Larrea, David Lawson, Ana Flavia Lucas d'Oliveira, Anna K-J Macintyre, Kerry MacQuarrie, Muriel Mac-Seing, Emily Ann Maistrellis, Shelly Makleff, Cicely Alice Marston, Crinda Marwah, Julia McReynolds-Perez, G J Melendez-Torres, Erica Millar, Kirstin Mitchell, Ismalia Z Mohammed, Sanjay K Mohanty, Abdu Mohiddin, Florence Muheirwe, Poonam Muttreja, Jefferson Mwaisaka, Denise Nacif Pimenta, Priya Nanda, Sharmishtha Nanda, Rishita Nandagiri, Nakkeeran Nanjappan, Francis Obare, Funmilola Morinoye OlaOlorun, Jeffrey O'Malley, Tricia Maria Ong, Bayla Ostrach, Sabu S Padmadas, Jitendra Pariyar, Lucia Berro Pizzarossa, Chelsea Polis, Tosin Popoola, Helen Potts, Davoud Pourmazi, Mizanur Rahman, Preety Rajbangshi, Mala Ramanathan, Anubha Rastogi, Juliet Richters, Anissa Rizkianti, Kathryn W Roberts, Sam Rowa, Ana Paola Ruiz, Malabika Sarker, Marta Schaaf, Erica Sedlander, Sumitra Sharma, Kristen Shellenberg, Aamod Dhoj Shrestha, Suzanne Sicchia, Holly Donahue Singh, Arushi Singh, Nelloy Sircar, Ilene S Speizer, Rachel Spitzer, Lara Stemple, Bianca Maria Stifani, Lucila Szwarc, Mildred Tambudzai Mushunje, Amanda Tanner, Siri Tellier, Jennifer Thomson, Jissa Vinoda Thulaseedharan, Mahhub Ul Alam, Verónica Undurraga, Boniface Ushie, Heini Väisänen, Cecilia Van Hollen, Ravi Verma, Leela Visaria, Krani Vora, Yohannes Wado, Megan Wainwright, Helen A Weiss, T Charles Witzel, Susan Yanow, Erick Kiprotich Yegon, Katherine Young, Bethany Young Holt, Phyu Phyu Thin Zaw

www.srhm.org / www.srhmjournal.org

Twitter @SRHMJournal

Facebook @SRHMJournal

